# Radiogenic heat production in the Coastal Plain Sands of Ipokia, Dahomey Basin, Nigeria

**DOI:** 10.1016/j.mex.2019.07.006

**Published:** 2019-07-08

**Authors:** T.A Adagunodo, O.G. Bayowa, M.R. Usikalu, A.I. Ojoawo

**Affiliations:** a Department of Physics, Covenant University, Ota, Nigeria; b Department of Earth Sciences, Ladoke Akintola University of Technology, Ogbomoso, Nigeria; c Department of Physics, University of Ibadan, Ibadan, Nigeria

**Keywords:** Radiometrics, Ifonyintedo, Dahomey Basin, Radiogenic heat production, Coastal Plain Sands, Thermal conductivity

## Abstract

This study is aimed at establishing the radiogenic heat production in the Benin Formation of Dahomey Basin using in-situ measurements of elemental concentrations of uranium, thorium and potassium over a kaolin deposits in Ifonyintedo, Ipokia LGA, Ogun State. Super-Spec gamma ray spectrometer is used to obtain varied data of naturally occurring radionuclides over eleven different stations in the Coastal Plain Sands at 1 m height above the ground. The mean heat produced by uranium, thorium and potassium are 294.3, 410.5 and 0.001 ρW kg^−1^ respectively. The total heat produced in the study area varied from 544.0 to 902.1 ρW kg^−1^, which classified the study area as Low-to-Moderate Heat Production Potential zone. This classification corresponds to the radiogenic heat characteristics of Benin Formation in Niger Delta.

• In-situ measurements of three radioisotopes were made over Coastal Plain Sands of Dahomey Basin.
• The mean heat produced by uranium, thorium, potassium and the total radiogenic heat are 294.3, 410.5, 0.001 and 704.79 ρW kg^−1^ respectively.
• The study area is characterized by Low-to-Moderate Heat Production Potential.

## Graphical abstract

**Figure:**
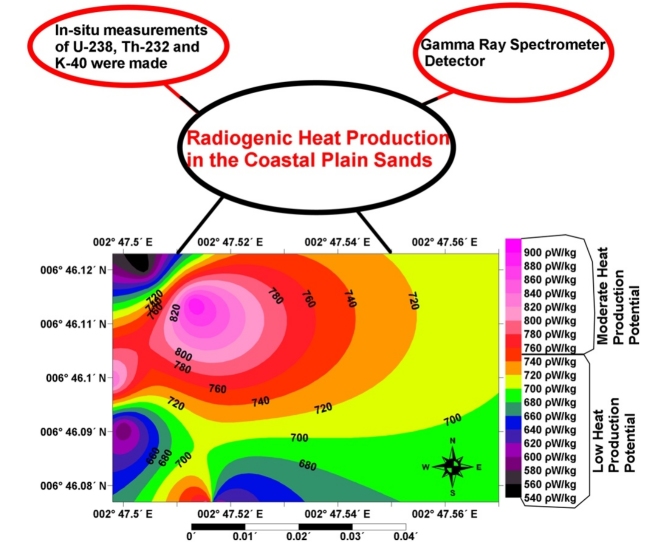

**Specifications Table**

**Table:** 

Subject Area:	Earth and Planetary Sciences
More specific subject area:	Applied Geophysics
Method name:	Radiometrics
Name and reference of original method:	Radiogenic heat production
Resource availability:	Super-Spec Gamma Ray Spectrometer; Surfer 11 Software

## Method details

The radioactive heat production (heat that arises from the decay process of long-lived radioactive elements), heat generated when the planet was accreted and formed (which is being cooled to date), and the frictional heating arising from the dense outer core, which sinks to the inner core are the three heat sources arising from the earth’s interior [1,2]. In radioactive or radiogenic heat production, the isotopes involved in this process are known as primordial or primeval radioactive elements. In Geophysics, it is believed that primordial isotopes are nuclides that are available on earth in abundance, which have been in existence as they are even before the Earth was formed [3].

Thermal conductivity, boundary conditions, sedimentation and erosion processes, water flow and radiogenic heat sources are the controlling factors of thermal structure in a sedimentary terrain. Heat production through radiogenic source varies in lithology, with shale being the major contributor to heat production [4]. In basin analysis, the required parameters for modeling of geodynamics state of a basin and thermal maturation of Oil-Source rocks include: thermal gradient, heat flow and radiogenic heat production [5]. It is also a key factor in trapping and sealing mechanism of hydrocarbon, such as diagenesis of sediments and fluid overpressure; for example diagenesis of sediments and fluid overpressure all depends on temperature.

Ogun state is one of the promising states to join the oil producing communities in Nigeria. The state is blessed with an oil-rich Island, which is situated at Tongeji and environs, Ipokia Local Government (LG). Other unexplored locations are believed to exist within Ipokia local government, which could be located either in offshore or onshore of the local government area as the state is richly blessed with kaolin clay minerals as well. Despite numerous applications of clay minerals such as kaolinite and chlorite among others in oil and gas exploration, relatively few works have been documented on the perspective of clay minerals in oil and gas exploration. This paper focuses on the radiogenic heat production from Oligocene to Recent kaolin deposits of the Dahomey Basin, and discusses its thermal implications on hydrocarbon generation in the study area.

### Geological setting of the study area

The study was conducted in Ifonyintedo, which is bounded by latitude 6.76795–6.76877 °N and longitude 2.79163–2.79283 °E, located in Ipokia LG, Ogun State. Ipokia LG is bounded by Yewa LG in the north, Badagry LG (of Lagos State) in the south, Yewa South and Ado-Odo/Ota LGs in the east, Republic of Benin in the west. Ipokia LG is strategically located as the major frontier between the Republic of Benin and Nigeria, which has contributed to the internal generated revenue of the LG to the Federal Government through Idiroko boarder. This has enabled them to fondly rename the LG as the main gate to the gateway state. The LG is blessed with the oil rich Tongeji Island and environs, a major investment opportunity waiting to be harnessed by both local and foreign investors. Ifonyintedo falls within a tropical climate zone, with rainy (March–November annually) and dry (November–March annually) seasons. The annual mean temperature in the study area is 26.5 °C [6].

**Fig. 1:**
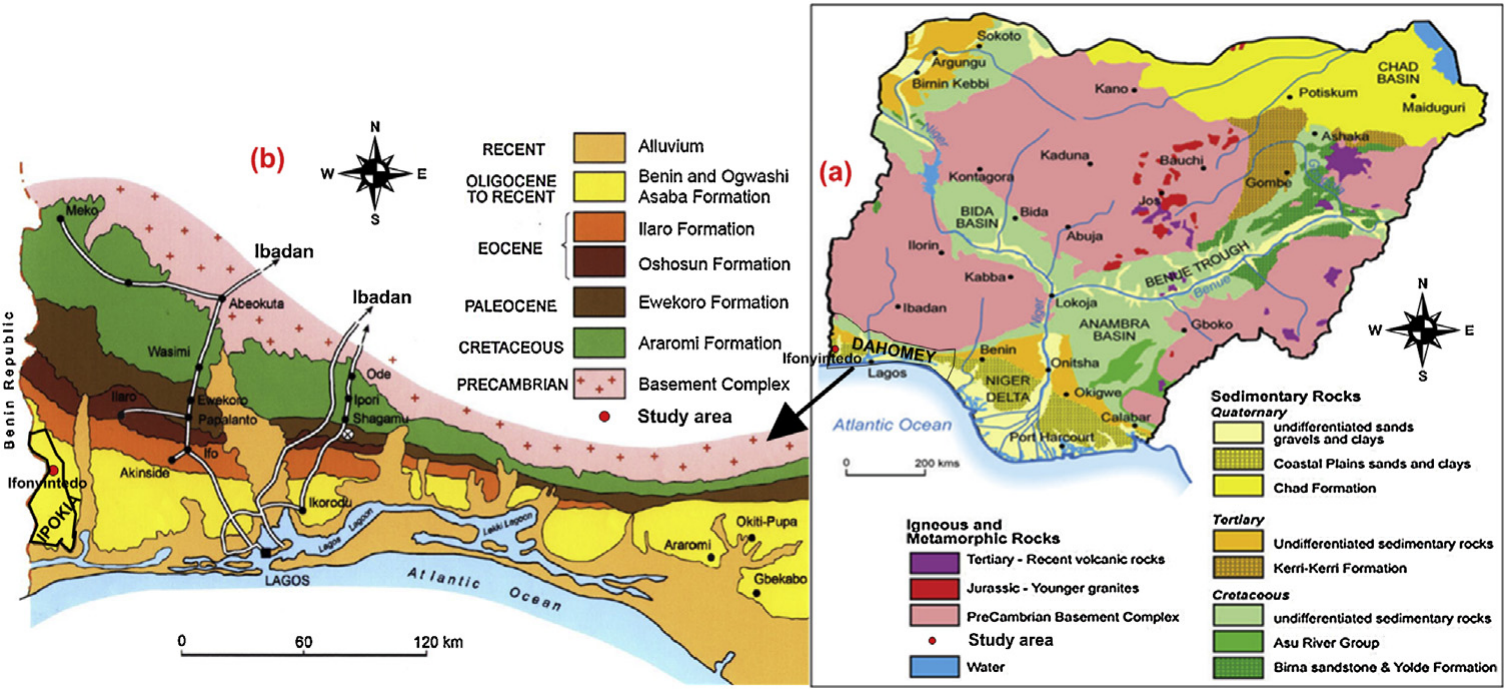
Geological maps of (a) Nigeria (b) Dahomey (Benin) Basin revealing the study area.

The Basement Complex and Sedimentary rocks are the major rocks that are believed to have covered Nigeria almost in equal proportions. The rocks of Basement Complex are considered to be PreCambrian in age [[7], [8], [9], [10]], while the sediments varied from Upper Cretaceous to Recent in age [6,[11], [12], [13]]. The Basement Complex rocks are composed of igneous and undifferentiated metamorphic rocks, as well as their weathering products (which are popularly known as overburden). The sedimentary rocks are classified into seven major Basins, which are: Anambra, Benue, Borno (Chad), Bida (Nupe or Mid-Niger Delta), Niger Delta, Dahomey (Benin) and Sokoto (Lullemeden) Basins [14] (Fig. 1). The study area is underlain by the rocks of Dahomey Basin, which are classified into six Formations and have been discussed in the literature [12,15]. These Formations include: the Alluvium; Benin Formation or Coastal Plain Sands; Ilaro Formation; Oshosun Formation; Ewekoro Formation; and Abeokuta Formation. Ifonyintedo is founded on the Coastal Plain Sands of Dahomey Basin, which is considered to span from Oligocene to Recent in age (Fig. 1).

The topography of Ifonyintedo is majorly characterized by undulating lowland, which slopes from N – S. This implies that all the major rivers in this axis (as well as the entire state) flow as tributaries into either Atlantic Ocean or Coastal Lagoons [16]. The major rivers that follow this flow pattern in Ogun State include: Ohu; Yewa; Oyan; Abafon; Awuko; Iju; Iseje; Ojumo among others. Kaolin clay is generally available across the study area, which is one of the important rocks in hydrocarbon exploration. Its applications include: tectonic evolution; thermal history and source rock maturation history; depositional environment; diagenetic history and prediction of reservoir quality; and generation, migration and accumulation processes in hydrocarbon [17].

### Materials and methods

A ground gamma survey to determine the concentrations of potassium (K), Uranium (eU) and thorium (eTh) was carried out in Ifonyintedo, Coastal Plain Sands of Eastern Dahomey Basin. The data were randomly taken at 11 different locations, such that it covered the upper terrain of kaolin deposits field in the study area using Super-Spec gamma ray spectrometer, model RS-125. The data collection were obtained at 1 m height above the ground, where the measurements at a spot were done four different times, in order to estimate the average of K, eU and eTh present in each location. RS-125 was manufactured by Canadian Geophysical Institute, with an integrated large detector, data storage, inbuilt algorithm to compute the data and display through its screen, high sensitivity and device measurement error of about 5%. The device is suitable to display the measured concentrations of K, eU and eTh in percentage (%) for K, and part per million (ppm) for eU and eTh respectively. Further description about RS-125 composition can be obtained in the report of [6]. This study is part of the mega 2017–2018 Geophysical-Radiometric campaign embarked on by our research team. Four isotopes have been identified as capable radiogenic elements to produce heat, these include: ^238^U; ^235^U; ^232^Th and ^40^K. The ^235^U isotope has shorter half-life and releases extra energy when undergoing it decay when compared to ^238^U. This enables ^238^U to be suitable isotope in generation of radiogenic heat. The radiogenic heat (Q) produced by rock radioactivity with concentrations of uranium (C_U_), thorium (C_Th_) and potassium (C_K_) can be determined by Eq. (1) as proposed by Rybach [17]. This equation has been as applied by [[18], [19], [20]]. Hence, the obtained radioactivity data were used to determine the radiogenic heat production in Ifonyintedo.

(1) Q = 0.00348 (C_K_) + 95.2 (C_U_) + 25.6 (C_Th_)

### Method descriptions

**Fig. 2:**
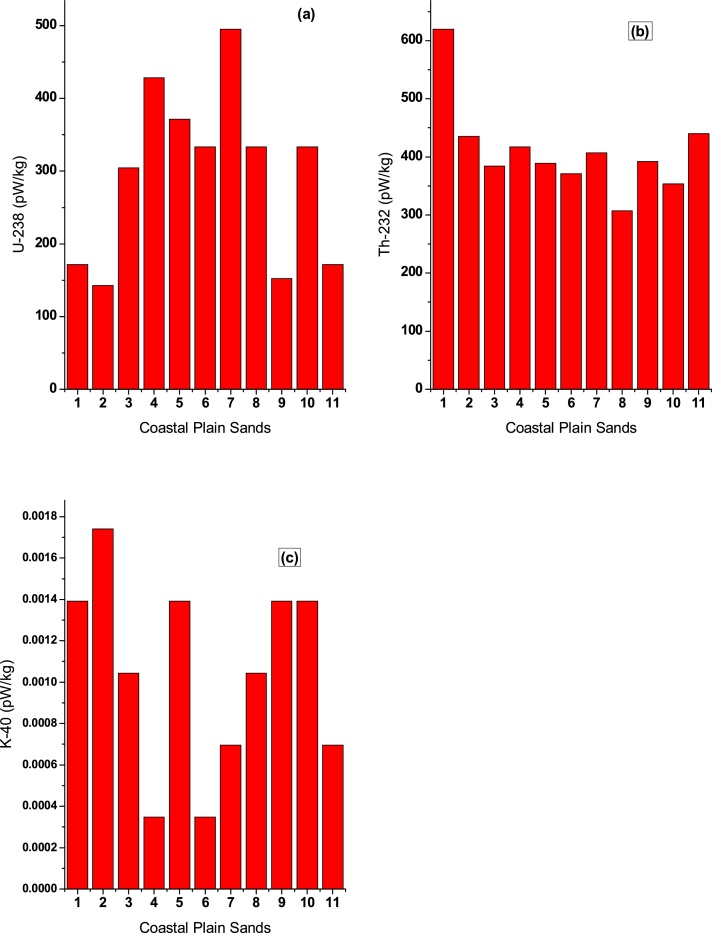
Contributions of heat produced by: (a) uranium (b) thorium (c) potassium.

The results obtained from study are presented as table, graphs and map. The radioisotope concentrations that is responsible for the heat production in the upper terrain of kaolin deposits in Ifonyintedo varied from 1.5 to 5.2 ppm for ^238^U; 12.0 to 24.2 ppm for ^232^Th; and 0.1 to 0.5% for ^40^K, with mean values of 3.1 ppm, 16.0 ppm and 0.3% for ^238^U, ^232^Th and ^40^K respectively as shown in Table 1. In the same view, the radiogenic heat produced by the three radionuclides in the Coastal Plain Sands of kaolin deposits in Ifonyintedo varied from 142.8 to 495.04 ρW kg^−1^ for ^238^U; 307.20 to 619.52  ρW kg^−1^ for ^232^Th; and 0.000348 to 0.001740 ρW kg^−1^ for ^40^K, with mean values of 294.3, 410.5 and 0.001 ρW kg^−1^ for ^238^U, ^232^Th and ^40^K respectively (Table 1 and Fig. 2). The total radiogenic heat production (Q) in the study area varied from 544.0 to 902.1 ρW kg^−1^, with a mean value of 704.8 ρW kg^−1^. This implies that the total heat production in the upper terrain of kaolin deposits in Ifonyintedo varied from Low-to-Moderate Heat Production Potential (HPP). This is in agreement with the classifications of heat production by [4], who categorized Q < 750 ρW kg^−1^ as Low HPP, 750 < Q < 1500 ρW kg^−1^ as Moderate HPP, and Q > 1500 ρW kg^−1^ as High HPP for radiogenic heat production.

**Table 1:** Radioactivity concentrations and heat produced by the three isotopes in the Coastal Plain Sands.

	Radioactivity concentrations			Heat produced per radioelement (ρW kg^−1^)			
Location	^238^U (ppm)	^232^Th (ppm)	^40^K (%)	^238^U	^232^Th	^40^K	Total heat produced (Q)
CPS 1	1.80	24.20	0.40	171.36	619.52	0.001392	790.88
CPS 2	1.50	17.00	0.50	142.80	435.20	0.001740	578.00
CPS 3	3.20	15.00	0.30	304.64	384.00	0.001044	688.64
CPS 4	4.50	16.30	0.10	428.40	417.08	0.000348	845.68
CPS 5	3.90	15.20	0.40	371.28	389.12	0.001392	760.40
CPS 6	3.50	14.50	0.10	333.20	371.20	0.000348	704.40
CPS 7	5.20	15.90	0.20	495.04	407.04	0.000696	902.08
CPS 8	3.50	12.00	0.30	333.20	307.20	0.001044	640.40
CPS 9	1.60	15.30	0.40	157.32	391.68	0.001392	544.00
CPS 10	3.50	13.80	0.40	333.20	353.28	0.001392	686.48
CPS 11	1.50	17.20	0.20	171.36	440.32	0.000696	611.68
Mean	3.09	16.04	0.30	294.25	410.53	0.001044	704.79
Range	1.5–5.2	12.0–24.2	0.1–0.5	142.80–494.04	307.20–619.52	0.000348–0.001740	544.00–902.08

Shales are majorly composed of clay minerals (kaolinite, illite and smectite) and quartz grain, which are known for its richness in organic matter and a key potential source rock in Sedimentary Basin [21,22]. It has also been noted for its high radionuclide contents when compared to other rock types [23,24]. This could have resulted to the Low-to-Moderate HPP as recorded in the study area, since the study was conducted on a kaolin deposits.

**Fig. 3:**
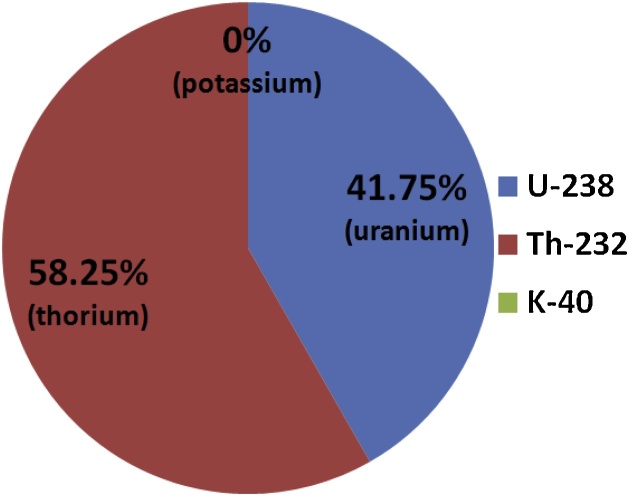
Percentage distributions of heat produced by each isotope.

The percentage distributions of radioelements in the study area showed that uranium and thorium contribute at 41.75 and 58.25% per radioelement to the total radiogenic heat production. As revealed in Fig. 3, contribution by potassium is negligible. Thorium is the highest radiogenic heat contributor in the Coastal Plain Sands of Dahomey Basin. This is in agreement with the work of [4], who used laboratory measurement to obtain the radiogenic heat production in the Cretaceous sediments of Benue Trough. In the same context, the outcome of this study supports that of [25], who employed Gamma Ray Spectrometer detector in the laboratory to examine the contributions of three basic radioelements to the radiogenic heat production in sediments of Ogun river, Nigeria. The report of [20] over a granitic terrain of FCT, Abuja presented uranium as the major contributor, while potassium is regarded as a trace element. This could have been attributed to geological composition of Abuja [26], since granitic rocks are the major intrusive bodies to Basement Complex in Nigeria [27]. Generally, granites are enriched in ^238^U and ^232^Th [28]. The ratio of ^232^Th to ^238^U could be high in some fractured or fault zones in Basement Complex terrain [10,29,30], while it could be low in some terrain where uranium has not undergone depletion due to lack of fluid infiltration [31], as revealed in the case of [20].

**Fig. 4:**
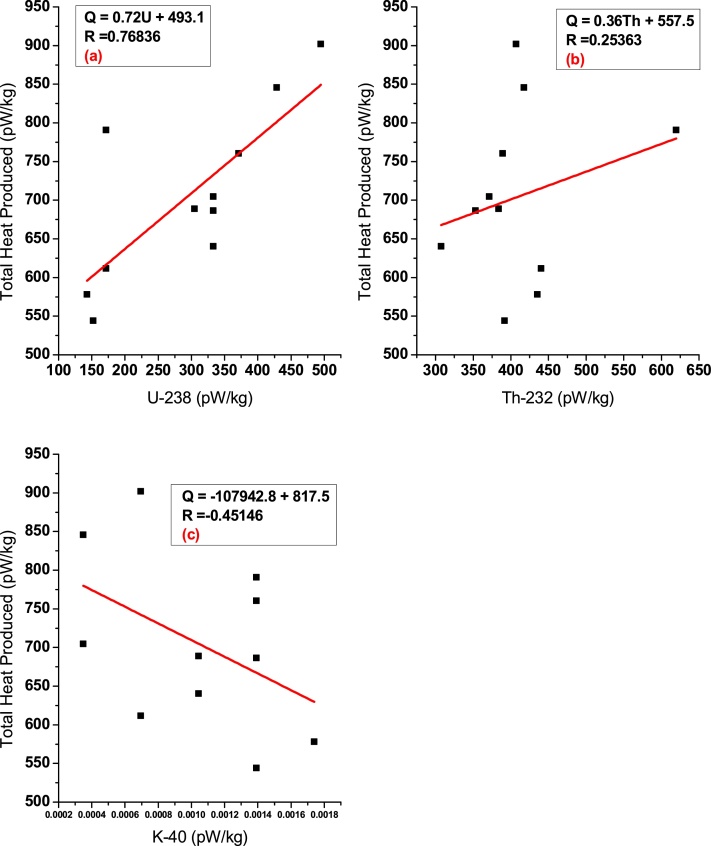
Relations between (a) uranium (b) thorium (c) potassium and the total heat produced in the Coastal Plain Sands.

**Fig. 5:**
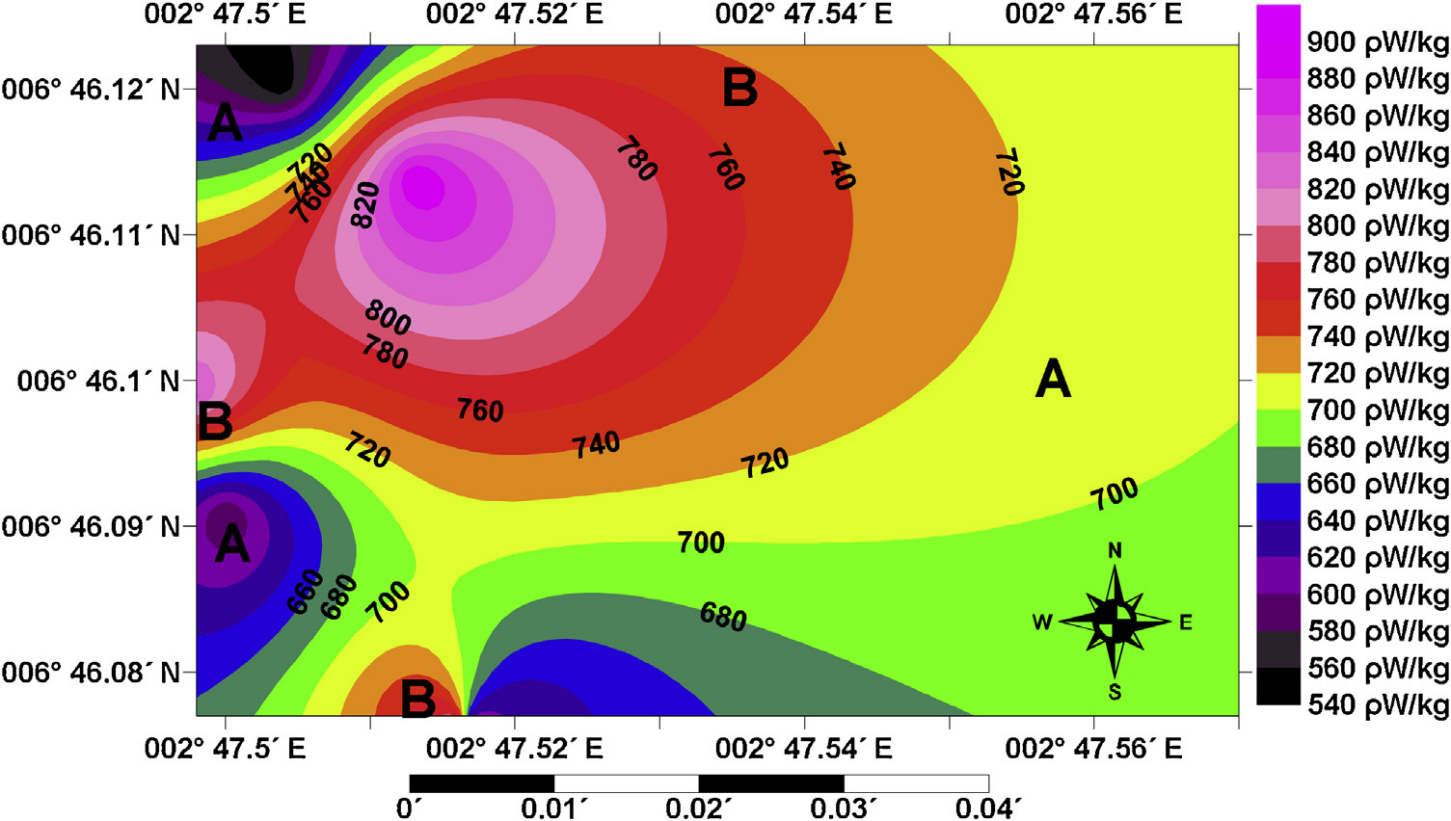
Composite map for the radiogenic heat production in the Coastal Plain Sands.

The linear relations between the radionuclides and the total heat produced in the study area are shown in Fig. 4. The linear models and the correlation coefficients (R) between Q and uranium, thorium and potassium are: Q = 68.5U + 493.1, with R = 0.76836 for ^238^U; Q = 9.2 Th + 557.5, with R = 0.25363 for ^232^Th; and Q = −375.6 K + 817.5, with R = −0.45146 for ^40^K respectively. Strong and very weak correlation coefficients exist in Fig. 4a between ^238^U and total heat produced, and in Fig. 4b between ^232^Th and total heat produced, respectively. This implies, the higher the uranium and thorium, the higher the total heat produced in the study area. In Fig. 4c, negative weak correlation coefficient is observed. This signifies that the lower the potassium, the higher the total heat produced. From Fig. 4, it is obvious that uranium and thorium contributions are from the same source (possibly from the parent rock of Dahomey Basin), while contributions of potassium to the total heat produced could be as a result of weathering effect on feldspar minerals that produced kaolin in the study area [6]. These models (Fig. 4) have revealed that no matter how small the geogenic contribution might be from any of these three basic radionuclides, they are of importance in the production of total radiogenic heat in the Coastal Plain Sands of Dahomey Basin. The composite map for the total radiogenic heat production in the study area is presented in Fig. 5. The map revealed that Low HPP (which is marked as A) constitutes the eastern, northeastern, southeastern, southern and some minor areas at the tips of southwestern and northwestern zones respectively. However, the Moderate HPP (which is marked as B) traverses from western through the central to the northern zones of the study area. The low HPP zones in the study area could be zones for uranium leaching, as uranium remains the major source of radiogenic heat in formation of kaolinite [32]. As reported by [33], kaolinite is the later product derived in hydrothermal decomposition (an active process in formation of kaolin) of feldspar. Therefore, the radiogenic heat pattern in Fig. 5 reveals that thermal regime is highly concentrated in the NW and SW axes of the study area.

## Conclusion

In this study, the estimated radiogenic heat production in the Coastal Plain Sands of Dahomey Basin varied from 544.0 to 902.1 ρW kg^−1^. This has classified the thermal regime of the study area (Dahomey Basin) as Low-to-Moderate HPP zone, which corresponds to the radiogenic heat characteristics of the topmost stratigraphy colum (Benin Formation or Continental Alluvium Sand) in Nigeria Delta Basin of Nigeria. Further downward probe within the crust could result to a Moderate-to-High HPP that is capable of producing oil and gas within the source rock in the study area.
